# “We communicate poorly with each other” - Registrars’ perceptions of communication skills training

**DOI:** 10.15694/mep.2021.000119.1

**Published:** 2021-05-10

**Authors:** Hester Alida Swart, Elize Archer

**Affiliations:** 1 Stellenbosch University

**Keywords:** Communication skills training, Registrars, Post-graduate training, communication skills curriculum development

## Abstract

This article was migrated. The article was marked as recommended.

Background

There is strong evidence that patient health outcomes are improved by effective clinician communication both with their patients and each other. Despite this, there is still a lack of training in and assessment of communication skills in postgraduate curricula in South Africa. When designing a communication skills training curriculum for registrars, their views and needs should be considered as they may impact the effectiveness of such an intervention.

Objectives

To explore registrars’ understanding of what constitutes effective communication in the workplace and the value thereof. Furthermore, the registrars’ communication skills training experiences and needs were determined.

Methods

This was an exploratory qualitative study conducted at a government hospital in Cape Town, South Africa. Registrars’ perceptions were explored making use of one on one interviews that were transcribed, coded, and themed using an inductive, iterative approach.

Results

Ten registrars working in the Department of Obstetrics and Gynaecology were interviewed individually. Registrars highly value effective communication skills and are of the opinion that they can improve with training. The participants experienced challenges when communicating with colleagues, particularly with consultants. The registrars made recommendations for the improvement of communication skills training at postgraduate level.

Conclusion

Registrars expressed a need for formal training to facilitate effective communication with colleagues, especially with consultants. These results should be considered when developing a postgraduate curriculum for communication skills training. Enhancing the quality of registrar-consultant interactions may have a positive influence on both the standard of patient care and registrars’ clinical learning environment.

## Introduction

It is widely accepted that effective doctor-patient communication is linked to better health outcomes ( Joosten *et al.*, 2008; Haskard Zolnierek and DiMatteo, 2009). Furthermore, there is evidence that poor communication between healthcare colleagues is associated with an increased rate of adverse patient outcomes and litigation ( Sutcliffe, Lewton and Rosenthal, 2004; Greenberg *et al.*, 2007).

Medical educators have responded with increased focus on the training of communication skills in both undergraduate and postgraduate medical programmes around the world ( Frank and Danoff, 2007; Swing, 2007). Although undergraduate medical curricula in South Africa (SA) have largely kept up with this trend, communication skills training and assessment at a specialist trainee level seems to have lagged behind ( Dacre *et al.*, 2004; Van Staden *et al.*, 2011; *The Colleges of Medicine of South Africa*, 2016). A contributing factor to this may be the paucity of research on postgraduate communication skills training in low and middle-income countries ( Scott *et al.*, 2016).

It is necessary to explore SA registrars’ views on the value of communication skills and their training in this field. This is especially important considering the challenges they face in the public healthcare sector ( Pepper, 2011). The results of this study should assist in the planning and implementation of a contextualised postgraduate communication skills training curriculum.

## Methods

### Study design

This was an exploratory research study utilising qualitative data.

### Study setting

The study was conducted at Tygerberg Hospital (TBH) which is a tertiary, government-funded public hospital in the Western Cape Province, SA. This hospital also serves as a postgraduate training centre in association with Stellenbosch University (SU).

### Study participants and recruitment

Inclusion criteria were that participants had to be specialist trainee registrars in the Department of Obstetrics and Gynaecology for at least 18 months. Purposeful sampling was used to invite registrars from different ages, genders, and cultural backgrounds ( Denscombe, 2014). The study was reviewed and approved by the SU Health Research Ethics Committee (HREC ref: S18/10/235) and institutional permission was granted by SU (IRPSD-1146). Furthermore, permission was granted in accordance with the Provincial Research Policy and Tygerberg Hospital Notice No 40/2009.

### Data collection

Individual in-depth interviews were conducted by the principle investigator (HAS) who is a specialist obstetrician and gynaecologist at TBH. Written consent was obtained from all participants. Recruitment continued until no significant new themes emerged from the data ( Denscombe, 2014). A total of 10 interviews were conducted with a mean length of 34 minutes each using a semi-structured interview guide with prompts. All interviews were audiotaped and transcribed verbatim by a professional transcription service after identifiers were removed.

### Data analysis

The transcribed interviews were thematically analysed ( Saldana, 2013). An inductive, iterative process was observed throughout the study. The interview data was coded by hand and grouped into categories from which themes and subthemes were identified *.* The transcripts and themes were reviewed by 5 participants and the supervisor to allow additional input to improve credibility. HAS kept a reflexive diary during the research process and considered how her assumptions may influence the data analysis ( Berger, 2015).

## Results/Analysis

### Sample characteristics

Interviews were conducted with 10 of the 33 (30.3%) registrars working in the Department of Obstetrics and Gynaecology. The median age of the participants was 36.2 years (IQR of 4.50 years), 80% were female, and their median training duration was 24 months (IQR of 13.25 months). Their gender and cultural background represented that of all the registrars in the department.

### Findings

The key themes and subthemes derived from the transcripts are shown in Table 1.

**Table 1:** List of themes and subthemes derived from the transcripts’ analysis

Code	Themes	Subthemes
C1	Registrars’ views on communication skills in general	1.1 Understanding of communication skills 1.2 Value of communication skills 1.3 Their own level of communication skills
C2	Communicating with colleagues	2.1 Communicating with all colleagues 2.2 Communicating with consultants 2.3 Implications of communication breakdown
C3	Postgraduate communication skills training practices	3.1 Quality of training 3.2 Approaches to training 3.3 Clinical learning environment
C4	Recommendations for communication skills training	

#### C1: Registrars’ views on communication skills in general

Although the registrars discussed how communication skills include both doctor-patient and inter-colleague communication, most registrars emphasised the component of communicating with colleagues.

‘My idea of communications when you asked me, it’s more like work colleagues, interpersonal work relationships.’ (R2)

It became clear that the registrars’ value both communication skills and training therein, especially in the context of their strenuous working conditions. One registrar discussed how the association between pressures on hospital resources and the increasing threat of litigation could complicate doctor-patient communication.

‘Sometimes it might be our fault because now there is only one theatre, there is a delay ... it’s difficult to convey that message. It’s like, oh, you are not as urgent as the other person, I have to cut her Caesar first, and now as a result, at the end, this baby died. I find it difficult to say the right thing so that the hospital can avoid getting legal action.’ (R2)

Evident during the interviews was that most registrars believed that the quality of their communication is acceptable, but that it could be improved by further training. By way of illustration:

‘I don’t think I’m the best communicator, but I think I learn, every day I learn something, and I’m surely not the same communicator I was a few years back.’ (R8)

#### C2: Communicating with colleagues

Registrars elaborated on their communication with consultants, peers, junior colleagues, nursing colleagues, and medical students. Their focus was more on the challenges of communicating with colleagues compared to communicating with patients.

‘I like patients. I easily sort of build a rapport with them ... I more easily communicate with patients, probably than more senior ‘up there’ people.’ (R4)

‘Because we also communicate very poorly with each other, and that’s I think very sad for the department because you don’t want to even ask a colleague for help.’ (R5)

Registrars elaborated on several ways in which communication between colleagues is problematic. One registrar mentioned how they perceive the communication between colleagues to be focused on finding fault.

‘I think that we are often, in medicine, very critical of each other, and the way that we communicate is not always very, it might come across as a bit judgemental in a way.’ (R7)

Furthermore, one registrar deliberated on the language diversity in the department and how the use of a specific language may make non-speakers feel excluded.

‘... but for some people who have no clue about Afrikaans, then it’s a bit of a challenge for them, because then they will feel left out ... it’s not a nice thing to be part of the team, but you’re not part of the conversation ... People do it in the tearoom all the time.’ (R10)

Several factors that contribute to poor communication with colleagues were discussed in the interviews. Many registrars perceive the multicultural environment they work in as a big challenge.

‘It’s much easier to relate to somebody that is from the same cultural background ... it’s just you may be able to communicate a bit better. It just maybe comes a bit more naturally.’ (R7)

Furthermore, a number of registrars felt that communication between colleagues is influenced by differences in character and fluctuations in mood.

‘I think we have enough proof that interpersonal skills are lacking [chuckles] ... I don’t know if a lot of it has to do with people, just how they are and their backgrounds and their personalities.’ (R4)

‘So, it doesn’t matter whether you are the best communicator, if you wake up on the wrong side of the bed, you are going to step on somebody’s toes.’ (R8)

Additionally, many registrars felt that the pressures of the working environment have a negative effect on the quality of communication.

‘So, I think under the ideal circumstances, I can communicate quite effectively, but I think what often happens is, due to overworking and fatigue and pressures of just load, it definitely suffers a bit.’ (R7)

Most registrars volunteered that they find it especially difficult to communicate with consultants compared to other colleagues.

‘So, I think there is quite big room for improvement as far as communication is concerned between for instance a consultant and a registrar ... I believe it that’s one area that we need to emphasise, both from the registrars’ point of view and also from the consultant point of view.’ (R10)

Registrars believed several factors contributed to their difficulty in communicating with consultants in particular. The impact of perceived power imbalances between them was mentioned by several registrars.

‘Very senior colleagues have very strict ways or ideas, and sometimes you can’t fully express your own opinion, you just have to go along with things. Like, you’re too scared to speak to some of the consultants.’ (R5)

Some registrars discussed how coming from different cultural backgrounds made it difficult for them to communicate freely within this perceived power-imbalance. By way of illustration:

‘culture also plays a role in that, because like in my culture, let’s say maybe Prof. Xfor example, I look at him as if he is my father. So, let’s say even if he shouts at me and I think that it’s unfair, I cannot approach him and say no Prof., what you did to me, it’s not fair, you weren’t supposed to say that. No, I can’t.’ (R6)

Several registrars discussed how poor communication between colleagues, including all levels of experience, impacts the continuity of patient care negatively.

‘So, the moment handing over people to people is a bit of a problem ... you will maybe get a phone call that the patient is on theatre table, and you know nothing about the patient, just because someone forgot ...’ (R4)

Furthermore, many registrars elaborated on their perception that poor communication with consultants has a negative impact on their clinical learning environment.

‘...and then there are other people who shove papers in your face while you’re on a ward round, in front of people, and say, “we’re going to take your reg job away if you don’t get this question right”.’ (R5)

#### C3: Postgraduate communication skills training practices

Registrars’ perception of the quality of the communication skills training they receive varied widely.

‘There is nothing [chuckles]. It’s like non-existent [laughs]. Sorry.’ (R4)

‘I think it is adequate. It’s good.’ (R7)

In general, the registrars had superficial insight into the differences between formal and informal training. After clarification of the definition, most registrars perceived their communication skills training to be largely informal.

‘I don’t think there is lots of formal training in terms of communication skills, but there’s lots of bedside training, and there’s lots of opportunity to witness other colleagues that have more experience in communicating.’ (R7)

Regarding informal training, the registrars acknowledged the value of both positive and negative role modelling. Some registrars perceived mostly negative role modelling.

‘I think there are more negative role models than positive ... so it’s a lot of: I don’t want to be like that.’ (R5)

Some registrars were concerned about feedback practices between consultants and registrars and how it is role modelled to them. Others perceived the feedback to focus mainly on their faults and that it is often delivered unnecessarily harshly.

‘...if people did something right, then compliment them on that, so that they are motivated to do it right. I think in our department people focus more on what people miss to do right, and I think that is not an effective communication.’ (R10)

One registrar discussed how feedback and role modelling as part of their informal training are often not seen and utilised as learning opportunities by trainees.

‘I think it’s easy to kind of, because it’s not formal, it’s easy to kind of just look past it, in a way, and not be open to it.’ (R7)

Some registrars mentioned that they perceive their clinical learning environment to be unsupportive at times.

‘I don’t know if it’s a good thing or a bad thing, but one thing I have learnt in the profession is where you are thrown in at the deep end of the pool, and then watch if you can swim or if you drown.’ (R8)

#### C4: Recommendations

Many registrars suggested that communication skills should be formally taught and assessed. They recommended that such training should include communication between colleagues.

‘One would have felt that maybe it should have been part of the syllabus, because in my training, it was just an informal thing. There was no exam written about it. There was no formal lecturing around it. But I’m sure it’s a gap that one needs to look at very closely.’ (R9)

Regarding content, registrars emphasised topics related to improving communication with colleagues. Many registrars highlighted the challenges of communicating with colleagues after an adverse event.

‘It was her, like I think first or second call ... there were two or three babies who passed away, and it’s not her fault. She literally had a breakdown in the labour ward, and nobody feels the need to talk to this person, or not the need, it’s just that nobody knew what to tell her. Even the consultant was like okay, let’s find someone more sensitive to talk to her ... I feel there is no one to teach you afterwards how to deal with the situation.’ (R2)

## Discussion

The registrars in this study perceived communication skills and its training to be of great importance. This view is reassuring, especially considering that their training seems to focus mainly on the biomedical aspects of patient care at the cost of so-called “soft-skills” ( Williams, Cantillon and Cochrane, 2001; Levinson, Lesser and Epstein, 2010). There is evidence that this focus is even more evident in the context of high patient load and limited resources as is mostly the case for SA registrars ( Razack *et al.*, 2007; Scott *et al.*, 2016).

Unexpectedly, the registrars emphasised communication challenges they experience with colleagues more than those with patients. This finding is similar to that of Luthy *et al.* ( Luthy *et al.*, 2004) where the registrars noted communication with colleagues as one of their major challenges. Registrars interviewed for the index study highlighted how the multicultural, multilingual environment and the high-pressure, low-resource setting in which they work has an important impact on communication. Additionally, they elaborated on how an individual’s character and mood may affect the way s/he communicates with colleagues. Excessive workload and a multicultural working environment are both factors identified in the literature that may negatively impact communication between colleagues ( Hall *et al.*, 2004; Flowerdew *et al.*, 2012).

On further exploration of communication practices between colleagues, registrars emphasised difficulties when communicating with consultants. The challenge of negotiating the complex relationship between registrars and consultants is well known ( Balmer, Giardino and Richards, 2012). Registrars discussed how they view the power imbalance and cultural differences between registrars and consultants to be important role players. The literature supports the notion that this power discrepancy may result in registrars being bullied and feeling afraid to report adverse events ( Scott, Blanshard and Child, 2008). Additionally, cross-cultural differences may affect registrars’ threshold to “speak up” when there is a difference of opinion with their consultants ( Kobayashi *et al.*, 2006).

Registrars further elaborated on the negative impact of poor communication amongst colleagues on the continuity and safety of patient care ( Sutcliffe, Lewton and Rosenthal, 2004). Additionally, the influence of poor communication between registrars and consultants on the clinical learning environment was discussed. Most experts agree that postgraduate medical education occurs largely in the form of experiential learning and apprenticeships in the workplace ( Teunissen *et al.*, 2007). In accordance with this, the registrars in this study perceived their own training in communication skills to be mostly informal in the form of role modelling and feedback.

The registrars made several recommendations on how their communication skills training could be improved. They suggested that it should be formally taught and assessed and emphasise communication skills between colleagues, particularly between registrars and consultants. One approach to address this need may be to implement a short, formal communication skills course during their first year of training. As part of this course, registrars could be taught how to better understand and utilise the mostly informal training they will be exposed to further. Although the focus thus far has been on interventions for registrars, communication challenges should be addressed on multiple levels including faculty development in the form of training programmes for consultants ( Bylund *et al.*, 2008).

There is a need to improve the formal training and assessment of registrars’ communication skills in SA. This study addressed the need to understand registrars’ perceptions of communication skills training. Without this valuable information, communication skills curricula may be designed that are inappropriate for our context.

### Study limitations and strengths

The individual in-depth interviews allowed the interviewer to explore social and personal matters, possibly on a deeper level compared to group interviews. This may have resulted in the registrars’ unexpected emphasis on the challenges of communicating with colleagues. Generalisability of this study is potentially restricted by the small study sample of registrars working in a single discipline. However, there is significant overlap in the contextual challenges that registrars working in different specialities and geographical areas face and thus shared training initiatives across disciplines are possible ( Razack *et al.*, 2007).

### Suggestions for further research

This needs analysis could be strengthened by exploring the views of more stakeholders for instance consultants, patients, and hospital managers. Furthermore, it would be valuable to assess the impact of implementing a short, formal communication skills training course for registrars early in their training.

## Conclusion

Registrars in this study identified a need for training in the skill of communicating with colleagues, particularly with consultants. This should be considered when planning the development of a postgraduate communication skills curriculum. Enhancing the quality of registrar-consultant interactions may have a positive influence on both the standard of patient care and registrars’ clinical learning environment.

## Take Home Messages

• Registrars experiences challenges when communicating with colleagues, particularly with consultants.
• Registrars expressed a need for formal training to facilitate effective communication with colleagues, especially with consultants.
• These results should be considered when developing a postgraduate curriculum for communication skills training.
• Enhancing the quality of registrar-consultant interactions may have a positive influence on both the standard of patient care and registrars’ clinical learning environment.

## Notes On Contributors

Dr. Hester Alida Swart. MBChB; FCOG(SA); MMed (O&G); MPhil (HPE). Specialist and senior lecturer at the Department of Obstetrics and Gynaecology, Faculty of Medicine and Health Sciences, Stellenbosch University, Tygerberg Campus.

Dr. Elize Archer. BSocSc (Nursing), BCur Hons (ICU), MPhil (Higher Education); PhD (Health Professions Education). Senior lecturer at the Centre for Health Professions Education, Faculty of Medicine and Health Sciences, Stellenbosch University, Tygerberg Campus.
