# Supplementary material for: “We communicate poorly with each other” - Registrars’ perceptions of communication skills training
Source: MedEdPublish (2016). 2021 May 10;10:119. [Version 1] doi: 10.15694/mep.2021.000119.1 (PMC10939521; doi:10.15694/mep.2021.000119.1)
Supplement: Peer review history

# Reviewer report: Reviewer response for version 1

**DOI:** 10.21956/mep.18920.r27046

**Published:** 2021-05-18

**Authors:** Leila Niemi-Murola

**Affiliations:** 1 University of Helsinki

This review has been migrated. The reviewer awarded 3 stars out of 5 This is an interesting article focusing on registrars’ perception of communication skills training. In my country, there is a structured program to train medical undergraduate students’ communication skills, but later in their careers they should seek formal education themselves. Communication is a competency included in many EPAs, but it is seldom trained as an entity. Thus, it was interesting to read the registrars perceptions about effective communication. The authors seem to have concentrated on verbal communication excluding written communication. It is understandable in a country having more than one official language, and the results are generalizable in countries having similar circumstances.It was interesting to read that the major communication problems were encountered in interaction with peers and consultants, because this interaction is often taken as self-evident and formal undergraduate education is focused on interaction with patients. This is an interesting finding which makes one reflect on trainees’ challenges in one’s own institution.

Reviewer Expertise:

NA

No decision status is available

# Reviewer report: Reviewer response for version 1

**DOI:** 10.21956/mep.18920.r27047

**Published:** 2021-05-12

**Authors:** Mary Higgins

**Affiliations:** 1 University College Dublin, National Maternity Hospital

This review has been migrated. The reviewer awarded 4 stars out of 5 I really enjoyed your paper and thank you for submitting it. I wished to make a couple of comments with the aim of clarification and possible improvement. 1. It is excellent that you performed member checking to confirm the results, but why include the supervisor?2. Fair dues on the reflexive diary - massively impressed3. As well as informal learning from experiential learning and apprenticeship, what about the Hidden Curriculum (which feeds into both I guess)4. Suggest you could check out the SRQR or COREQ standards - but you seem to have met most of the SQRQ standards already.

Reviewer Expertise:

NA

No decision status is available
